# Increased Salivary BDNF and Improved Fundamental Motor Skills in Children Following a 3-Month Integrated Neuromuscular Training in Primary School

**DOI:** 10.3390/jfmk9030154

**Published:** 2024-08-30

**Authors:** Fidanka Vasileva, Raquel Font-Lladó, Gemma Carreras-Badosa, Víctor López-Ros, Anna Ferrusola-Pastrana, Abel López-Bermejo, Anna Prats-Puig

**Affiliations:** 1 Pediatric Endocrinology Research Group, Biomedical Research Institute of Girona, 17190 Girona, Spain; fidankavasileva@gmail.com (F.V.); ge.carreras@gmail.com (G.C.-B.); alopezbermejo@idibgi.org (A.L.-B.); 2 University School of Health and Sport, University of Girona, 17190 Girona, Spain; raquel.font@udg.edu (R.F.-L.); aferrusola@euses.cat (A.F.-P.); 3 Faculty of Education and Psychology, University of Girona, 17004 Girona, Spain; victor.lopez@udg.edu; 4 Research Group of Culture, Education and Human Development, Institute of Educational Research, University of Girona, 17004 Girona, Spain; 5 Chair of Sport and Physical Education—Centre of Olympic Studies, University of Girona, 17004 Girona, Spain; 6 Department of Biology, University of Girona, 17003 Girona, Spain; 7 New Therapeutic Targets Group, Department of Medical Sciences, Faculty of Medicine, University of Girona, 17071 Girona, Spain; 8 Department of Medical Sciences, University of Girona, 17071 Girona, Spain; 9 Pediatric Endocrinology, Dr. Josep Trueta Hospital, 17007 Girona, Spain; 10 Research Group of Clinical Anatomy, Embryology and Neuroscience, Department of Medical Sciences, University of Girona, 17071 Girona, Spain

**Keywords:** children’s health, locomotor skills, control and manipulative skills, exercise intervention, physical education, saliva

## Abstract

Brain-derived neurotrophic factor (BDNF) is a protein involved in synaptic transmission and neuronal plasticity, which underlie the processes of learning and memory formation. Acute exercise and exercise training increase BDNF concentration. We aimed to evaluate the effects of a 3-month integrated neuromuscular training (INT) on salivary BDNF concentration and the mastery of fundamental motor skills in school-aged children. An additional goal was to explore the associations between potential changes in BDNF and fundamental motor skills. Sixty-seven primary school-aged children were randomly allocated to control (N = 32; 7.52 ± 0.31 y) or INT groups (N = 35; 7.56 ± 0.29 y). A 3-month INT was applied during the warm-up of physical education (PE) classes, twice weekly. Salivary BDNF was measured using a sandwich-enzyme-linked immunosorbent assay and the mastery of fundamental motor skills was assessed using the CAMSA test, at baseline and after 3 months. The children in the INT group, as compared to the children in the control group, exhibited higher salivary BDNF (F = 8.865; *p* = 0.004), higher scores for sidestep (F = 13.240, *p* = 0.001), 1-foot hop (F = 11.684, *p* = 0.001), kick (F = 4.010, *p* = 0.050), the sum of locomotor skills (F = 18.799, *p* < 0.0001), and the sum of control and manipulative skills (F = 8.151, *p* = 0.006), as well as the total sum of fundamental motor skills (F = 11.266, *p* = 0.001) after the 3 months. Interestingly, the increase in salivary BDNF concentration after the 3-month INT was associated with an improvement in locomotor skills (beta = 0.385; *p* = 0.039; adjusted R^2^ = 0.088) and the total improvement in fundamental motor skills (beta = 0.428; *p* = 0.020; adjusted R^2^ = 0.124). A school-based 3-month INT increased salivary BDNF and improved the mastery of fundamental motor skills in children, highlighting the positive impact of this intervention for a pediatric population.

## 1. Introduction

Brain-derived neurotrophic factor (BDNF) belongs to the family of neurotrophins and it is a protein encoded by the BDNF gene [1,2]. This protein is secreted from various tissues such as the brain, skeletal muscle, cardiac muscle, lungs, thymus, liver, spleen, and immune system cells [3,4]; it has also been found in blood and saliva [4,5]. BDNF has various functions in the human body, but it is mainly considered as a key protein involved in the regulation of the nervous system function [1,2,3]. It promotes the survival of existing neurons and encourages the growth and differentiation of new neurons and neuronal synapses [1,2]. Moreover, BDNF is involved in modulating synaptic transmission, as well as facilitating long-term potentiation and neuronal plasticity, all of which underlie the processes of learning and memory formation [1,2]. Higher BDNF levels have been related to better attention, memory, and cognitive performance [6]. Interestingly, an increase in BDNF concentration has been observed after both acute and chronic physical exercise [6,7,8,9,10,11,12,13,14,15,16]. For instance, a high-intensity exercise bout induced an immediate increase in serum and plasma BDNF in young adults [6]. Endurance training induced a notable increase in serum and plasma BDNF in healthy humans, as well as patients with multiple sclerosis [7]. An intense resistance exercise and a 3-month cross-fit training intervention increased serum BDNF in young adults [14,16]. A 40 min continuous exercise and a high-intensity intermittent exercise increased the secretion of serum BDNF in patients with type 1 diabetes [15]. However, some studies reported unchanged BDNF concentration after exercise [17,18].

Integrated neuromuscular training (INT) is a specialized exercise program with a progressive load that improves fitness, fundamental motor skills, and overall health [19,20,21]. These programs mainly consist of exercise combinations designed to improve neuromuscular function and often incorporate resistance training, plyometrics, coordination, dynamic stabilization, and speed and agility drills [19,20,21]. The goal of INT applied to athletes is to increase their capacity to perform complex movements efficiently, potentially leading to improved sports performance and reduced risk of injury [19]. Additionally, when applied to pediatric population, INT has been recognized as an effective strategy for enrichment of the motor learning experience in children [20,21].

Even though various definitions exist, in general, fundamental motor skills refer to the individual’s basic capacity to perform and efficiently solve various motor tasks while engaging in sport, physical activities, or even everyday life activities [22,23]. Among the most studied fundamental motor skills are locomotor, control, and manipulative skills, which are essential for performing activities of daily living, as well as representing a basic foundation for physical activities and sport [21,22,23,24]. Hence, these skills are especially important for each individual and crucial for their growth and motor development [21,22,23]. Locomotor skills enable individuals to move from one place to another and include walking, running, jumping, hopping, leaping, galloping, sliding, and skipping [21,22,23]. Control and manipulative skills refer to the capacity for handling and manipulating objects (e.g., ball, hoop, rope, etc.) and they include throwing, catching, dribbling, kicking, striking, and underhand rolling [23,25]. These skills are crucial for successful participation in any kind of physical and sports activities [26].

It is important to note that the fundamental motor skills are developed during childhood, and then, continuously improved throughout life [22,27,28,29,30,31]. More precisely, it has been established that most children develop fundamental motor skills and reach acceptable levels of quality until the age of 6, so an “open window” for improvement of the mastery of fundamental motor skills appears at the age of 7 [22,27,29,30,32]. Indeed, childhood has been referred to as a “golden era”, i.e., a critical period, decisive for the development and improvement of the mastery of fundamental motor skills, by many authors [22,27,30]. During this critical period, the brain’s ability to change and adapt in response to various experiences is higher, thus children can develop strong neuronal synapses and acquire skills faster than later in life [30,33]. As children grow, they experience continuous modifications in their fundamental motor skills due to a complex interplay between neuromuscular maturation, previous motor experiences, and the variety of new motor experiences that they encounter [27]. These continuous modifications in children’s fundamental motor skills lead to improvements in the mastery of fundamental motor skills [27]. Indeed, the diverse motor experiences that children are exposed to induce a reorganization of the neural pathways in the brain and formation of new neuronal synapses, which are processes that are mediated by BDNF [1,2,27]. The acquisition of high levels of mastery of fundamental motor skills is especially important for several reasons [22,27]. First, it enables children to successfully cope with the challenges of everyday life, to interact with the environment, as well as to adapt easily to a changing environment [27]. Then, it has important health implications and it is considered crucial for children’s physical, social, and psychological development [22]. Interestingly, fundamental motor skills may also serve as a foundation for an active lifestyle because previous evidence showed a relationship between higher levels of fundamental motor skills and higher levels of physical activity [22,27]. And lastly, research showed positive associations between fundamental motor skills and academic advancements, as well as general well-being [22].

Because previous studies that applied a 3-month exercise intervention reported a significant increase in BDNF concentration [13,16], we hypothesized that a 3-month INT applied in primary schools may potentially induce increased BDNF concentration in the saliva of healthy children. We focused on salivary BDNF to avoid blood extraction, which may be painful and traumatic, especially for children, and mainly because previous studies have already demonstrated the utility of saliva to assess BDNF concentration [5,34]. Additionally, we hypothesized that the 3-month INT would induce improvements in the mastery of fundamental motor skills of these children, because previous INT interventions applied either in sports clubs or school environments were effective in improving sport-specific skills, as well as fundamental motor skills [19,35,36].

Taking into consideration previous research, the objective of the present study is to evaluate the effects of a 3-month INT implemented in primary schools on salivary BDNF concentration, and the mastery of fundamental motor skills in children. An additional goal was to explore the associations between potential changes in BDNF and fundamental motor skills.

## 2. Materials and Methods

### 2.1. Population and Ethics

Before initiating the study, the GRANMO 7.12 program was used to estimate the required sample size for inclusion considering a previous study that included protein quantification [37]. Accepting an alpha risk of 0.05 and a beta risk of 0.2 in a two-sided test, the total sample size for the present study was estimated to be 60 participants, i.e., 30 participants in each group.

A total of 67 apparently healthy children (34 boys and 33 girls; 7.5 ± 0.3 years) were recruited from schools in Cassà de la Selva and Salt (Girona, northeastern Spain). Both schools were in the same province and country, thus ensuring the homogeneity of the curricular content for the physical education (PE) classes, equipped with a multisport playground. Schools were randomly allocated either to the control (N = 32) or the INT group (N = 35), because randomization of children within the same school and during the same PE class was not possible due to the obvious ethical reasons. To prevent any perception of discrimination and ensure fair treatment for all participating children, the INT was offered to the control school upon study completion. Inclusion criteria were (1) no evidence of chronic or acute illness in the month preceding potential enrollment; and (2) age between 7 and 9 years. Exclusion criteria were (1) major congenital abnormalities; (2) illness or chronic use of medication; (3) musculoskeletal, neurological disorder and/or medication therapy that could alter postural stability and cardiorespiratory function; and (4) attending fewer than 80% of the PE classes. The present research was approved by the Institutional Review Board of Dr. Josep Trueta Hospital, Girona, Spain (CEIm:2016.134), and signed consent was obtained from the parents of all participating children. Prior to the start of the study, the PE teacher from the INT school was familiarized with the intervention and trained to deliver the INT sessions together with the researcher expert in INT [21]. All measurement procedures and sample collection were conducted on the same day, following previous familiarization with the test protocols.

Note that the control and the INT group were homogeneous in terms of the children’s sleep quality, physical activity levels, dietary habits, and the socio-economic status of their families. In order to control for the previously mentioned potential confounders, we collected accelerometer data (Triaxial Actigraph GT3X; detailed protocol is presented elsewhere [38]), and the children’s parents completed the following: (1) the KIDMED questionnaire for nutritional assessment [39]; (2) the PAU-7S questionnaire to obtain information on physical activity outside school [40]; and (3) an additional survey encompassing questions on the family’s socio-economic status.

### 2.2. Intervention

The frequency of the PE classes in the control as well as in the INT group was twice per week with a duration of 60 min. The PE classes had the following structure: an introductory segment (20 min), a main segment (30–35 min), and a concluding segment (5–10 min). Note that the main and the concluding segments were the same in both groups but the introductory segment was different.

The introductory segment for the control group consisted of a regular warm-up protocol, i.e., activities and games designed to progressively increase the heart rate and the range of motion in particular joints that would be predominantly engaged during the class [41]. On the other hand, the warm-up in the INT group consisted of implementation of the INT for 3 months. The INT covered 24 sessions organized in progressive circuits and games including strength, coordination, dynamic stabilization, plyometrics, speed, and agility exercises [21]. The sessions were delivered by a previously trained teacher and the researcher expert in INT [21]. After completion of the 20 min warm-up, the children continued with the main segment of the class.

The main segment of the PE classes for both groups was based on the curricular content outlined in the national curriculum for PE, which was didactically delivered by the teacher: (1) aerobic activities (running, jumping a rope) and activities that involve solving motor tasks in environmental conditions (outdoor circuits and polygons, orienteering activities); (2) activities designed to develop fundamental motor skills and abilities (motor challenges that contain elements from individual sports: athletics, gymnastics, tennis); (3) activities designed to develop interaction skills and teamwork (cooperative motor challenges that contain elements from team sports: football, basketball, handball, volleyball); (4) traditional and contemporary dances; and (5) outdoor activities in the natural environment (hiking, cycling, rollerblading, skating).

Finally, during the concluding segment of the PE classes, children from both groups engaged in light-intensity activities to cool down gradually. They could also ask questions, reflect on what they had learned during the class, and prepare for the next class.

### 2.3. Biological Samples Collection

Saliva samples were collected in the morning between 8:00 and 10:00 a.m. in a fasting state and stored at −20 °C according to the manufacturer’s protocol. Note that participants were asked to abstain from drinking water and brushing their teeth prior to collection. They were instructed to release 1–4 mL of saliva into a 5 mL polystyrene specimen tube after natural accumulation in the oral cavity. This procedure was repeated twice, at baseline and after 3 months.

### 2.4. Anthropometric Measurements

Anthropometric measurements were performed in the morning between 8.00 and 10.00 a.m. Body mass was measured with a calibrated digital scale (Portable TANITA, 240MA, Amsterdam, The Netherlands). Participants were instructed to stand barefoot on the Tanita 240MA platform wearing light clothes. Height was measured with a wall-mounted stadiometer (SECA SE206, Hamburg, Germany). Participants were instructed to stand barefoot with their heels against the backboard. BMI was calculated as body mass in kg divided by the square of height in m. Age- and sex-adjusted standard deviation scores (SDSs) for body mass, height and BMI were calculated using regional normative data [42]. All measurements were performed twice, at baseline and after 3 months.

### 2.5. Fundamental Motor Skills Assessment

Fundamental motor skills were assessed with the CAMSA test, which evaluates children’s capability to manifest, organize, and combine fundamental motor skills into structured movement patterns in a complex dynamic environment [43]. Note that the CAMSA test has also been validated in Spanish children (Supplementary Figure S1) [44]. The assessment considers both the quality of performance and the time spent to complete the task. To conduct the CAMSA test, we needed a wide non-slip surface, a tape, 6 hoops, 6 marker cones, 1 ball, 1 softball, and a wall target. Before initiation of the assessment, the 20 m polygon circuit was prepared and the wall target was marked (a square that is 61 cm wide and 46 cm high) according to the test protocol described by Longimur et al. (2017) [43]. The goal was to complete a 20 m polygon circuit effectively and as fast as possible while performing three 2-foot jumps, a 5 m right sidestep, 5 m left sidestep, one 2-handed catch, 1 precision overhand throw, a 5 m skip, six 1-foot hop right/left side, and 1 precision kick. Each child performed two practice trials followed by two measured trials, which were video recorded and the time was measured manually with a stopwatch. To ensure the integrity and blinding of the assessment procedure, the video recordings were made and coded by a researcher who was always different from the researcher expert who performed the final evaluation [21]. Fundamental motor skills were evaluated according to the criteria proposed by Longimur et al. (2017), and were grouped into [43] (1) locomotor skills (2-foot jump, sidestep, skip, 1-foot hop); and (2) control and manipulative skills (catch, overhand throw, kick). The CAMSA total score was also calculated as the sum of the scores obtained for each skill (maximum 14 points) and the time to complete the polygon circuit (maximum 14 points). Children who effectively combined speed and skill components received the highest CAMSA total score (maximum 28 points). The better result out of the two video-recorded trials (higher score) was considered for analysis. The intra-assessor and intra-subject CVs were <6%. There was no inter-assessor variability because all evaluations were performed by the same researcher expert in fundamental motor skills assessment [21].

### 2.6. Protein Concentration Quantification

BDNF was assessed in saliva with the Human BDNF ELISA kit (MBS355324; Gentaur, Spain), employing the sandwich enzyme-linked immunosorbent assay method. Initially, samples were defrosted at room temperature after being frozen for 2 years following saliva collection. Then, assay components and standards were prepared according to the manufacturer’s instructions. Subsequently, standards and saliva samples (100 μL per sample) were loaded onto the pre-coated microplate wells and incubated as indicated in the kit’s protocol. Following the indicated incubation periods, the wells were washed and the detection reagent was added. Finally, the reaction was stopped as indicated in the protocol, the absorbance was measured at the specified wavelength (450 nm) using a microplate reader, and the BDNF concentration was calculated using the log–log curve. The lower detection limit was 0.82 pg/mL and intra- and interassay CVs were <4%. BDNF concentration was conveniently quantified in all saliva samples and there were no outliers.

### 2.7. Statistical Analysis

Data were analyzed using the statistical software SPSS version 22.0 (SPSS Inc., Chicago, IL, USA). The normality of the data distribution was examined through the Kolmogorov–Smirnov test. Non-Gaussian variables were logarithmically transformed to improve the distribution symmetry. Prior to the intervention and after the random allocation of schools to the control or INT group, a *t*-test, Mann–Whitney U test, and chi-squared test were applied to ensure that the control and the INT group were comparable at baseline. To evaluate the effects of the 3-month INT on salivary BDNF and fundamental motor skills, we performed analyses of covariance by establishing general linear models that adjusted for potential confounding variables such as age, sex, BMI, and baseline values. Similarly, to explore the associations between potential changes in BDNF and fundamental motor skills, multiple linear regression analyses adjusting for the confounding variables were performed.

## 3. Results

Baseline characteristics and comparison between the control and the INT group are presented in Table 1. Based on Table 1, the study groups were comparable because there were no statistically significant differences between them in any of the studied parameters (*p* = 0.863 to *p* = 0.066).

In Table 2, we present the children’s characteristics after 3 months, as well as a comparison between the control and the INT group adjusted for age, sex, BMI, and baseline values. There were no significant differences between the two groups in terms of anthropometric characteristics: body mass (F = 0.131, *p* = 0.718); body height (F = 0.308, *p* = 0.581); and BMI (F = 0.221, *p* = 0.639). Interestingly, we observed a significant difference between the control and the INT group in salivary BDNF concentration (F = 8.865, *p* = 0.004; Table 2). The present results indicate that the 3-month INT induced a higher increase in salivary BDNF as compared to the traditional PE classes. Furthermore, we observed significant differences between the children from the control and the INT groups in terms of mastery of fundamental motor skills (Table 2). More precisely, children who performed the 3-month INT, in comparison to the children who had the traditional PE classes, showed greater improvement and higher scores for sidestep (F = 13.240, *p* = 0.001), 1-foot hop (F = 11.684, *p* = 0.001), kick (F = 4.010, *p* = 0.050), the sum of locomotor skills (F = 18.799, *p <* 0.0001), and the sum of control and manipulative skills (F = 8.151, *p* = 0.006), as well as the total sum of fundamental motor skills, i.e., CAMSA total score (F = 11.266, *p* = 0.001). Note that there were no significant differences between the two groups in the time to perform the test (F = 2.581, *p* = 0.114), 2-foot jump (F = 1.853, *p* = 0.179), catch (F = 3.357, *p* = 0.073), overhand throw (F = 3.283, *p* = 0.076), and skip (F = 3.808, *p* = 0.056). Overall, these results indicate that the 3-month INT was more effective than the traditional PE classes in improving the mastery of fundamental motor skills among children.

In Table 3, we show the associations between the changes in salivary BDNF concentration and fundamental motor skills after the 3-month period in both groups. There were no significant associations between the changes in salivary BDNF concentration and fundamental motor skills in the control group (beta = −0.039 to beta = 0.273; *p* = 0.196 to *p* = 0.851; adjusted R^2^ = −0.009 to adjusted R^2^ = 0.123). However, in the INT group, the increase in salivary BDNF concentration after the intervention was associated with the improvement in locomotor skills (beta = 0.385; *p* = 0.039; adjusted R^2^ = 0.088) and the total improvement in fundamental motor skills (beta = 0.428; *p* = 0.020; adjusted R^2^ = 0.124). Moreover, the present results indicate that the improvement in locomotor skills after the 3-month INT applied in primary school explained 9% of the variance of the increased salivary BDNF concentration after the intervention, whereas the total improvement in fundamental motor skills explained 12% of the variance of the increased salivary BDNF concentration after the 3-month INT.

## 4. Discussion

The main findings of the present study indicate that the 3-month INT implemented in primary schools increased salivary BDNF and improved the mastery of fundamental motor skills in children, thus highlighting the positive impact of this school-based intervention for the pediatric population. Moreover, the improvement in locomotor skills and the total improvement in fundamental motor skills after the intervention were related to the increased salivary BDNF concentration after the 3-month INT.

To the best of our knowledge, this is the first study assessing the effects of a 3-month INT on salivary BDNF concentration in children, thus it should serve as a starting point for future research. In general, the physiological mechanisms underlying the exercise-induced BDNF secretion are numerous and not completely understood, but one of them appears to be related to the exercise-induced increase in neuronal activity [45]. It is widely known that the sensory receptors send signals to the central nervous system during exercise through the afferent neurons, and subsequently, the efferent neurons transmit signals from the central nervous system to the muscles to initiate motor responses, thus resulting in increased neuronal activity [45,46]. The increased neuronal activity, thereafter, may potentially lead to increased BDNF secretion [45]. Considering this previously proposed mechanism for exercise-induced increase in BDNF secretion, one could speculate that the INT sessions, encompassing a variety of exercises, may have potentially induced an increase in the neuronal activity, including increased synaptic transmission, which subsequently may have led to increased BDNF secretion [1,2,45,46]. However, we should note that in the present study we did not assess neuronal activity, thus the proposed mechanism should be considered as a hypothetical explanation based on previous findings [1,2,45,46]. In addition to that, it seems to be far more complex because there is still a discrepancy in the scientific evidence with regard to the effects of physical exercise on BDNF secretion. In fact, numerous studies evaluating the effects of acute exercise or exercise training on BDNF concentration reported increased BDNF after the exercise session or the intervention [6,7,8,9,10,11,12,13,14,15,16], but there are also some studies that did not report exercise-induced changes in BDNF concentration [17,18]. A plausible explanation for the inconsistent observations has been proposed by Wang et al. (2022), who, after performing an extensive systematic revision with a meta-analysis, suggested that one potential reason for the different outcomes in the literature may be the difference in dose parameters [12]. Along these lines, the findings of the present study, indicating an INT-induced increase in salivary BDNF concentration, align with the findings of previous studies which showed that an exercise intervention with a duration of 3 months, i.e., the same duration as the intervention in the present study, significantly increased BDNF concentration [13,16]. Therefore, we suggest that a 3-month INT intervention may be sufficient to induce changes in BDNF secretion in school-aged children, which allows us to accept the first hypothesis. Considering that the present findings are in line with previous findings, we believe that the underlying mechanisms for the INT-induced increase in BDNF secretion should be further explored. We propose future studies conducted in laboratory settings to introduce electroencephalographic assessments or magnetic resonance imaging, with the aim of investigating the neuronal activity during INT and clarifying the present findings.

Furthermore, we also observed improvements in the mastery of fundamental motor skills after the 3-month INT, allowing us to accept the second hypothesis of the present study. There are probably various mechanistic explanations for the INT-induced improvements in fundamental motor skills [21,23,36,47]. One of them could be the process of learning, which is highly dependent on the quality and quantity of practice, i.e., the exposure to different motor experiences [21,23]. More precisely, the repeated exposure to basic motor patterns in complex dynamic settings stimulates muscle proprioceptors and joint mechanoreceptors to induce central and peripheral neuronal adaptations, which lead to a variety of responses, and that may potentially contribute to the improvement of the mastery of fundamental motor skills [36,47]. Along these lines, the 3-month INT is a specifically tailored intervention that consists of multiple sessions including various motor tasks in different environmental settings, offering a broad range of distinct motor experiences [21]. In support of our findings indicating INT-induced improvements in the fundamental motor skills among children, previous studies assessing the effects of INT reported similar outcomes [21,35,36,48]. For instance, a recent systematic revision assessing the effects of school-based neuromuscular training showed that neuromuscular training programs are superior in the development of fundamental motor skills as compared to traditional PE classes [36]. Then, a 10-week INT applied to children from primary schools significantly improved locomotor skills, as well as control and manipulative skills [48]. Another study that assessed the effectiveness of a 10-week INT applied in youth female volleyball players also reported significant improvement in locomotor skills, with large effects for lateral jumps [35]. Finally, note that we observed significant changes when considering the sum of locomotor skills, the sum of control and manipulative skills, and the total sum of fundamental motor skills in the present study; however, certain skills did not show significant changes when considered independently. Since, as previously discussed, the quantity of practice may impact the development of fundamental motor skills [21,23], we propose INT interventions with longer duration in the future, with the aim of potentially inducing more prominent changes in each fundamental motor skill independently.

Considering the increase in salivary BDNF concentration after the 3-month INT and the simultaneous improvement in fundamental motor skills, we further explored the potential associations between them. Interestingly, the improvement in locomotor skills and the total improvement in fundamental motor skills after the intervention were related to the increased salivary BDNF concentration in children who performed the 3-month INT in the school. Moreover, the improvement in locomotor skills explained 9%, whereas the total improvement in fundamental motor skills explained 12% of the variance of the increased salivary BDNF concentration in the children from the INT group. Studies exploring the direct associations between fundamental motor skills and salivary BDNF concentration in school-aged children are scarce. Also, the nature of the present study does not allow us to completely describe the mechanisms underlying the observed associations, but we can try to propose plausible explanations based on the present and previous findings. As previously discussed, we believe that the improvement in locomotor skills, or fundamental motor skills in general, may have resulted from the continuous exposure to various motor experiences and the increased quality and quantity of practice during the INT [21,23]. Furthermore, the continuous exposure to diverse motor experiences may have induced neuronal reorganization and formation of new neuronal synapses, which are processes mediated by BDNF [1,2,27]. Therefore, we hypothetically propose that the mechanistic link that could potentially explain the associations observed in the present study could be the exposure to various motor experiences, because on the one hand it may lead to improved mastery of fundamental motor skills, and on the other hand it may induce neuronal reorganization and formation of new neuronal synapses which are BDNF mediated [1,2,21,23,27,45]. In support of the findings in school-aged children observed in the present study, a previous study reported similar associations in older adults [49]. More precisely, the authors of the previous work found a positive association between the functional performance and plasma BDNF concentration in older individuals [49]. Since the observed associations in school-aged children seem to be in line with previous findings, we believe that future studies should be designed in laboratory settings with the aim of investigating further the proposed mechanisms and reinforcing the findings of the present study.

### 4.1. Limitations and Future Research

The present study has certain limitations which should be considered and potentially overcome in future research.

The major limitation of this study is the short duration of the INT intervention. We suggest future studies to focus on assessing the effects of INT with longer duration, compare the effects of INT with short and long durations, and finally, identify an optimal duration that will potentially induce more prominent changes in the mastery of fundamental motor skills.

We did not collect information on teaching quality in the present study, so we believe that future studies could address this issue by introducing surveys or questionnaires for teaching quality assessment, focus groups, interviews, etc.

While cerebrospinal fluid is considered to be the gold standard for assessment of central BDNF levels, and blood is also often used in research, in the present study we worked with saliva due to its less invasive nature and mainly because previous studies have already shown the utility of saliva for BDNF assessment [5,34]. However, saliva and even blood may not perfectly reflect central BDNF levels. Therefore, we believe that further studies in adult populations or in animal models should compare BDNF concentration in the cerebrospinal fluid and saliva, and examine to what extent saliva may reflect central BDNF levels. This could be especially relevant for sensitive populations such as the pediatric population.

Even though there were no statistically significant differences in salivary BDNF concentration between the control and the INT groups at baseline, the concentrations were not identical, thus the statistical analysis was adjusted for baseline levels. Indeed, in research with human populations, as is the case in the present study, a knockdown of the BDNF gene with the aim of obtaining two completely identical groups is not possible due to the obvious ethical reasons. Therefore, we propose the design of further studies in animal models to overcome this limitation.

Furthermore, we must consider that the present study is an interventional study which focused on short-term outcomes. In other words, we assessed the effects of a 3-month INT, but we did not assess the long-term effects of the INT intervention and their permanence. Thus, we encourage future research to employ a longitudinal design and investigate if INT-induced changes in terms of increased BDNF secretion and improved mastery of the fundamental motor skills are maintained over time, especially after a certain period from the completion of the INT intervention.

Also, schools were randomly assigned as a control or INT school in the present study but randomized group allocation of children within the same school and during the same PE class was not possible due to ethical reasons. Considering this, we believe that further studies assessing the effects of INT should be conducted in clinical settings. These studies could recruit school-aged children in primary health centers instead of schools, and apply the intervention as an extracurricular activity outside the schools, with the aim of allowing randomized group allocation of children.

Even though the control and the intervention groups were homogeneous in terms of children’s sleep quality, physical activity levels, dietary habits, and the socio-economic status of their families, there might have been certain heterogeneity in terms of sports practiced as an extracurricular activity. Future studies should try to overcome this potential limitation.

Finally, in the present study we assessed the effects of a 3-month INT considering two time points of measurement (baseline and after 3 months), which is the most common experimental approach for interventional studies. However, we believe that this traditional experimental approach may be complemented in the future with experimental designs that include analysis of long-time-series data on neuromuscular interactions. These studies may offer a direct monitoring of the entire process of neuromuscular interaction during the INT sessions, which will help researchers to better understand the changes induced by these interventions. Also, it would be worthwhile if further studies could be conducted in laboratory settings. These studies could introduce electroencephalographic assessments or magnetic resonance imaging, with the aim to investigate the neuronal activity during INT.

### 4.2. Practical Applications

Taking into consideration the various limitations of the present study, the current findings could mainly serve as a starting point for future research. Additionally, these findings may potentially encourage the incorporation of INT contents during PE classes because the INT seems to have a positive impact on the pediatric population. These findings may also guide PE teachers and exercise professionals when preparing the content for classes and training sessions that are aiming to improve the mastery of fundamental motor skills in children.

## 5. Conclusions

A 3-month INT implemented in primary schools as a warm-up activity during the PE classes increased salivary BDNF concentration and improved the mastery of fundamental motor skills in children. Moreover, the improvement in locomotor skills and the total improvement in fundamental motor skills after the intervention were related to the increased salivary BDNF concentration after the 3-month INT.

The present findings highlight the positive impact of this school-based intervention on the pediatric population.

## Figures and Tables

**Table 1:** Participants characteristics and comparison between the control and the INT groups at baseline.

Participant Characteristics at Baseline	Control Group (N = 32)	INT Group (N = 35)	*p*-Value
Age (years)	7.52 ± 0.31	7.56 ± 0.29	0.537
Sex (m/f)	16/16	18/17	0.144
Sleep (hours/day)	10.35 ± 2.05	9.62 ± 1.29	0.089
Physical activity (hours/day)	1.45 ± 0.93	1.85 ± 0.78	0.153
KIDMED (score)	6.61 ± 2.23	7.03 ± 2.04	0.448
Parental profession (non-degree/degree profession/NA)	2/25/5	8/26/1	0.091
Body mass (kg)	26.03 ± 3.33	27.32 ± 5.58	0.188
Body mass SDS	−0.28 (−0.67–0.10)	−0.23 (−0.78–0.22)	0.135
Height (cm)	126.22 ± 6.05	126.44 ± 5.22	0.862
Height SDS	−0.08 (−0.52–0.73)	−0.01 (−0.55–0.67)	0.682
BMI (kg/m^2^)	16.33 ± 1.44	16.82 ± 2.19	0.269
BMI SDS	−0.40 (−0.71–0.04)	−0.21 (−0.82–0.21)	0.251
BDNF (pg/mL)	116.58 (30.67–315.54)	327.69 (266.17–379.70)	0.154
Fundamental motor skills at baseline			
CAMSA time (s)	21.22 ± 4.36	21.77 ± 4.47	0.171
CAMSA 2-foot jump (score)	3.75 ± 0.57	3.86 ± 0.36	0.066
CAMSA sidestep (score)	2.00 (1.00–3.75)	1.00 (0.01–3.00)	0.327
CAMSA catch (score)	1.28 ± 0.85	1.00 ± 0.86	0.154
CAMSA overhand throw (score)	2.31 ± 1.59	2.20 ± 1.39	0.748
CAMSA skip (score)	2.00 (0.01–4.00)	3.00 (1.50–4.00)	0.142
CAMSA 1-foot hop (score)	2.00 (1.25–3.00)	2.00 (0.01–2.00)	0.272
CAMSA kick (score)	3.00 (1.00–4.00)	2.00 (1.00–3.00)	0.209
CAMSA locomotor skills (score)	10.06 ± 3.06	9.88 ± 3.83	0.819
CAMSA control and manipulative skills (score)	5.09 ± 2.90	4.12 ± 2.35	0.102
CAMSA total (score)	15.16 ± 3.67	13.53 ± 4.53	0.094

Data for Gaussian variables are presented as mean ± standard deviation. Data for non-Gaussian variables are presented as median and interquartile range. The *p*-value for Gaussian variables is from *t*-test. The *p*-value for non-Gaussian variables is from Mann–Whitney U test. The *p*-value for categorical variables is from chi-squared test. Significance level is set at 0.05. BMI: body mass index; INT: integrated neuromuscular training; KIDMED: questionnaire for assessment of dietary habits; NA: not available; SDS: standard deviation score.

**Table 2:** Participant characteristics and comparison between the control and the INT groups after 3 months.

Participant Characteristics after 3 Months	Control Group (N = 32)	INT Group (N = 35)	F	*p*-Value
Body mass (kg)	26.47 ± 3.64	27.44 ± 4.94	0.131	0.718
Body mass SDS	−0.29 (−0.64–0.16)	−0.14 (−0.58–1.20)	0.311	0.579
Height (cm)	127.67 ± 6.12	128.19 ± 5.51	0.308	0.581
Height SDS	0.04 (−0.48–0.89)	0.19 (−0.34–0.89)	0.831	0.365
BMI (kg/m^2^)	16.18 ± 1.44	16.66 ± 2.13	0.221	0.639
BMI SDS	−0.50 (−0.84–0.12)	−0.23 (−0.89–0.08)	0.074	0.786
BDNF (pg/mL)	138.38 (30.67–370.89)	406.78 (106.63–457.40)	8.865	**0.004**
Fundamental motor skills after 3 months				
CAMSA time (s)	19.87 ± 3.45	19.19 ± 2.53	2.581	0.114
CAMSA 2-foot jump (score)	3.71 ± 0.86	3.82 ± 0.46	1.853	0.179
CAMSA sidestep (score)	2.87 ± 1.81	4.45 ± 2.01	13.240	**0.001**
CAMSA catch (score)	1.27 ± 0.73	1.52 ± 0.67	3.357	0.073
CAMSA overhand throw (score)	2.47 ± 1.47	2.58 ± 1.25	3.283	0.076
CAMSA skip (score)	2.93 ± 1.53	3.61 ± 0.84	3.808	0.056
CAMSA 1-foot hop (score)	2.16 ± 1.21	2.70 ± 1.06	11.684	**0.001**
CAMSA kick (score)	2.45 ± 1.45	2.71 ± 1.27	4.010	**0.050**
CAMSA locomotor skills (score)	11.70 ± 3.04	14.58 ± 2.99	18.799	**<0.0001**
CAMSA control and manipulative skills (score)	5.33 ± 2.80	5.77 ± 2.44	8.151	**0.006**
CAMSA total (score)	16.42 ± 5.02	18.53 ± 5.02	11.266	**0.001**

Data for Gaussian variables are presented as mean ± standard deviation. Data for non-Gaussian variables are presented as median and interquartile range. Note that in the case of non-Gaussian variables, the log value was used in the analysis of covariance. The analysis of covariance is adjusted for age, sex, BMI, and baseline values. Significance level is set at 0.05 and significant values are marked in bold. BMI: body mass index; INT: integrated neuromuscular training; log: logarithmic; SDS: standard deviation score.

**Table 3:** Regression analyses representing the associations between the changes in fundamental motor skills and BDNF concentration.

	BDNF Δ %					
	Control Group (N = 32)			INT Group (N = 35)		
	Beta	*p*-Value	Adjusted R Squared	Beta	*p*-Value	Adjusted R Squared
CAMSA time Δ %	−0.159	0.413	0.022	0.152	0.446	−0.046
CAMSA 2-foot jump Δ %	−0.112	0.565	0.008	0.068	0.731	−0.061
CAMSA sidestep Δ %	−0.292	0.203	0.123	−0.154	0.560	0.046
CAMSA catch Δ %	−0.059	0.807	0.009	0.082	0.772	−0.056
CAMSA overhand throw Δ %	0.273	0.237	0.069	−0.210	0.449	−0.050
CAMSA skip Δ %	0.065	0.803	−0.043	0.196	0.379	−0.033
CAMSA 1-foot hop Δ %	0.171	0.426	0.030	−0.054	0.804	−0.045
CAMSA kick Δ %	0.098	0.661	−0.009	0.194	0.375	0.021
CAMSA locomotor skills Δ %	−0.213	0.268	0.044	0.385	**0.039**	0.088
CAMSA control and manipulative skills Δ %	−0.039	0.851	−0.015	0.101	0.621	−0.057
CAMSA total Δ %	−0.249	0.196	0.061	0.428	**0.020**	0.124

Adjusted for age, sex, and BMI. Significance level is set at 0.05 and significant values are marked in bold. BDNF: brain-derived neurotrophic factor; INT: integrated neuromuscular training; Δ %: percentage change (post-value versus pre-value).

## Data Availability

The original contributions presented in the study are included in the article. However, further inquiries for any additional data can be directed to the corresponding author upon a reasonable request.

## Supplementary Materials

The following supporting information can be downloaded at: https://www.mdpi.com/article/10.3390/jfmk9030154/s1.
