# Supplementary material for: Increased Salivary BDNF and Improved Fundamental Motor Skills in Children Following a 3-Month Integrated Neuromuscular Training in Primary School
Source: J Funct Morphol Kinesiol. 2024 Aug 30;9(3):154. doi: 10.3390/jfmk9030154 (PMC11417929; doi:10.3390/jfmk9030154)
Supplement: Supplementary file 1 [file jfmk-09-00154-s001.zip › jfmk-3154207-supplementary.pdf]

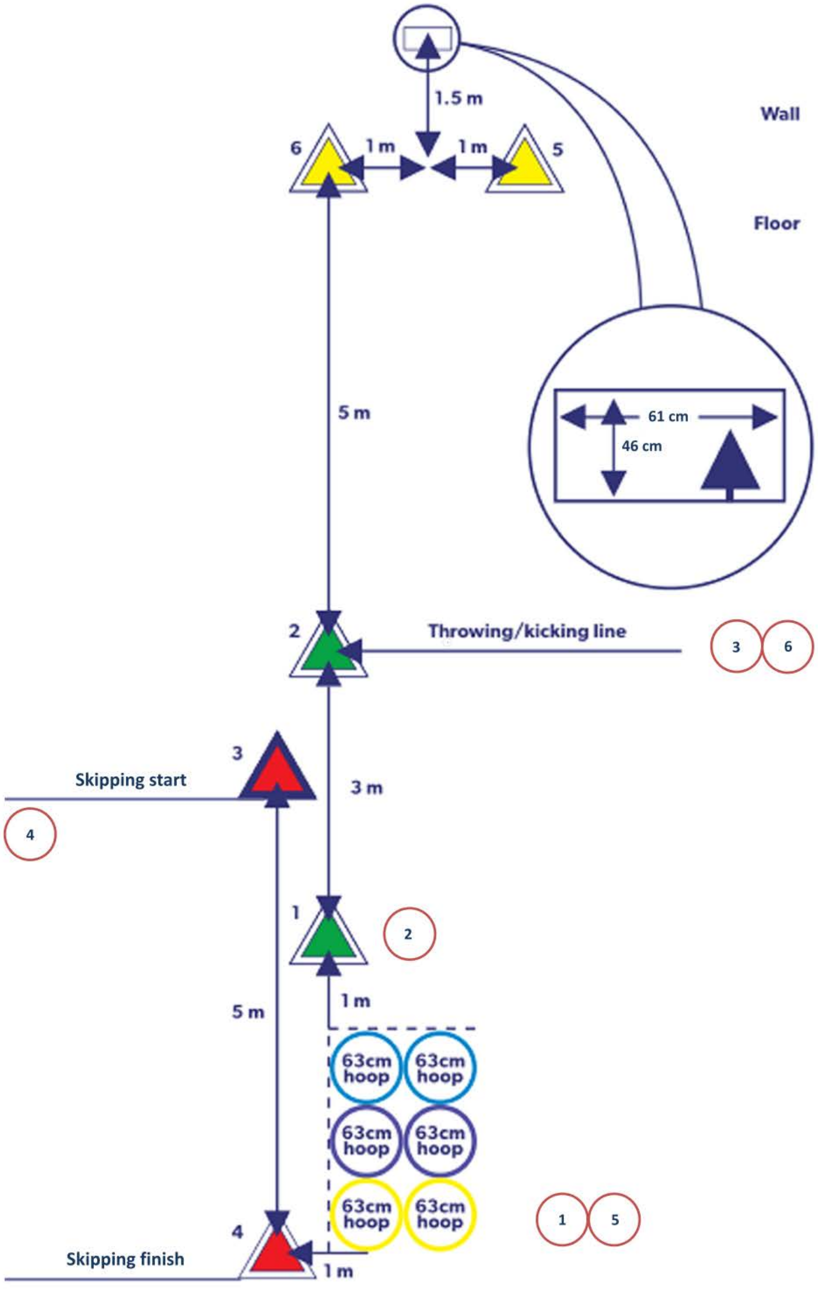

Supplementary Figure S1. CAMSA protocol was adopted by Longimur et al. (2017) and validated in Spanish children by Menescardi et al. (2022). The children were instructed to position themselves behind the hopes and to initiate the test on the signal given by the researcher in charge. The goal was to complete a 20 m polygon circuit effectively and as fast as possible while performing the required motor tasks. The children were constantly guided through the test by the researcher in charge with the following instructions: 1) ''Start jumping through the hopes while performing three 2-foot jumps''; 2) ''Slide sideways from cone 1 to cone 2 and then go back to cone 1''; 3) ''Catch the ball with both hands and then throw it overhand onto the wall target''; 4) ''Skip from cone 3 to cone 4''; 5) ''Hop through the hoops while performing six 1-foot hops''; and 6) ''Kick the ball onto the wall target''. Each child performed two practice trials followed by two measured trials, which were video-recorded and the time was measured manually with a stopwatch.

1. Longmuir PE, Boyer C, Lloyd M, et al. Canadian Agility and Movement Skill Assessment (CAMSA): Validity, objectivity, and reliability evidence for children 8-12 years of age. *J Sport Heal Sci*. 2017;6(2):231-240. doi:10.1016/J.JSHS.2015.11.004
2. Menescardi C, Villarrasa-Sapiña I, Lander N, Estevan I. Canadian Agility Movement Skill Assessment (CAMSA) in a Spanish Context: Evidences of Reliability and Validity. *Meas Phys Educ Exerc Sci*. 2022;26(3):245-255. doi:10.1080/1091367X.2021.2020794
